# Value of neutrophil-to-lymphocyte ratio for diagnosing sarcopenia in patients undergoing maintenance hemodialysis and efficacy of Baduanjin exercise combined with nutritional support

**DOI:** 10.3389/fneur.2023.1072986

**Published:** 2023-02-21

**Authors:** Jun Wang, Mei-chang Xu, Li-juan Huang, Bei Li, Lei Yang, Xu Deng

**Affiliations:** ^1^Nephrology Department of Nanjing Hospital of Integrated Traditional Chinese and Western Medicine, Nanjing, Jiangsu, China; ^2^Nephrology Department of Nanjing Hospital of Traditional Chinese Medicine, Nanjing, Jiangsu, China

**Keywords:** sarcopenia, neutrophil-to-lymphocyte ratio (NLR), Baduanjin exercise, nutritional support, maintenance hemodialysis

## Abstract

**Objective:**

To investigate the value of neutrophil-to-lymphocyte ratio (NLR) for diagnosing sarcopenia in patients undergoing maintenance hemodialysis (MHD) and efficacy of Baduanjin exercise combined with nutritional support on MHD patients with sarcopenia.

**Methods:**

A total of 220 patients undergoing MHD in MHD centers were selected, among which 84 had occurred with sarcopenia confirmed by measurements from the Asian Working Group for Sarcopenia. Data were collected for analyzing the influencing factors that lead to the onset of sarcopenia in MHD patients with the use of one-way analysis of variance and multivariate logistic regression. The role of NLR in the diagnosis of sarcopenia was explored, and its correlation with relevant diagnostic measurement performance such as grip strength, gait speed and skeletal muscle mass index was analyzed. In the end, some 74 patients with sarcopenia that qualify for further intervention and observation standards were divided into observation group (Baduanjin exercise plus nutritional support) and control group (nutritional support only), which were both intervened for 12 weeks. A total of 68 patients finished all interventions, with 33 patients in the observation group and 35 in the control group. The grip strength, gait speed, skeletal muscle mass index as well as the NLR were compared between the two groups.

**Results:**

With the employment of multivariate logistic regression analysis, it was found that age, hemodialysis duration and NLR were risk factors for the onset of sarcopenia in MHD patients (*P* < 0.05). The area under ROC curve for NLR of MHD patients with sarcopenia was 0.695, and NLR was negatively correlated with a biochemical indicator—human blood albumin (*P* < 0.05). NLR was also negatively correlated with patient's grip strength, gait speed and skeletal muscle mass index, with the same correlation found in sarcopenia patients (all *P* < 0.05). After intervention, patient's grip strength and gait speed were both higher, and the NLR lower in the observation group than those in the control group (*P* < 0.05).

**Conclusion:**

The occurrence of sarcopenia in MHD patients is associated with patient's age, hemodialysis duration and NLR. Therefore, it has been concluded that NLR has certain values in the diagnosis of sarcopenia in patients undergoing MHD. Moreover, the muscular strength can be enhanced and inflammation decreased in sarcopenia patients through nutritional support and physical exercise, i.e., Bajinduan exercise.

## Introduction

Maintenance hemodialysis (MHD) is one of the essential alternative treatments for end stage renal disease (1). Previous reports have shown that micro-inflammatory state, loss or insufficient intake of proteins as well as metabolic acidosis in patients undergoing MHD could lead to changes in muscle structure and decrease in muscular strength, ultimately resulting in sarcopenia (2). It has been reported that the prevalence rate of sarcopenia in MHD patients reaches as high as 3.9–63.3% (3, 4). Studies in China and some other countries around the world have suggested that sarcopenia could elevate the occurrence of several adverse events, thus impacting the prognosis of patients undergoing MHD (5, 6). According to some reports, the occurrence rate of sarcopenia in MHD patients and factors that contribute to the disease vary in different regions (7, 8). A study in recent years suggested that inflammation was closely associated with the onset of sarcopenia, as pro-inflammatory factors were significantly higher in patients with sarcopenia than those without (9). Neutrophil-to-lymphocyte ratio (NLR) is a novel inflammatory indicator that reveals the presence or absence of systemic inflammation in human bodies. Studies have shown that NLR plays a significant role in the occurrence and development of arteriosclerosis and tumors (10, 11). A previous report also demonstrated that NLR in MHD patients was related to Protein Energy Wasting, an important factor contributing to the onset of sarcopenia (12). One study reported that systemic inflammation could lead to decreased protein turnover and imbalance of cell growth, which further led to injury in skeletal muscles (13). It was also reported that the occurrence of sarcopenia was in close association with malnutrition and muscular hypoactivity (14). Studies outside China have indicated that treatments for sarcopenia could be realized through improving muscular mass and functions. Nutritional support as well as exercises are two effective approaches in treating this disease in patients undergoing MHD (15, 16). Therefore, based on the conclusions mentioned above, we investigated the diagnostic value of NLR in MHD patients, and the efficacy of Baduanjin exercise plus nutritional support on the treatment of sarcopenia in patients undergoing MHD.

## Data and methods

### General data

A total of 220 patients who received MHD in the MHD center of Nanjing Hospital of Integrated Traditional Chinese and Western Medicine and Nanjing Hospital of Traditional Chinese Medicine from October 2021 to May 2022 were selected. According to the diagnostic standards of Asian Working Group for Sarcopenia, 84 patients were confirmed with sarcopenia, among which only 74 were qualified for further clinical interventions. The 74 patients were randomized into different cohorts to receive nutritional support and Baduanjin exercise, namely, observation group for nutritional support plus Baduanjin exercise and control group for nutritional support only. During the intervention process, two patients from the control group and two from the observation group discontinued the trial because they were transferred to the other hospital, 2 from the observation group were not followed up any more for they were intolerant to the treatment. At last, the observation group included 33 patients and the control group 35. This trial was approved by the Ethics Committee of Nanjing Hospital of Integrated Traditional Chinese and Western Medicine and Nanjing Hospital of Traditional Chinese Medicine (No.: 2021035), with written informed consent obtained from all enrolled patients.

### Inclusion and exclusion criteria

Patients were eligible for the study if they conformed to the diagnostics standards of end-stage renal disease, their hemodialysis duration lasted for over 6 months, aged not < 18 years old and conformed to the diagnostic standards of sarcopenia by Asian Working Group for Sarcopenia (14), which were as follows: i. grip strength assessment: male should have grip strength no < 28 kg and female no < 18 kg, otherwise their muscular strength is considered as weakened; ii. gait speed assessment: if the walking speed of patients is no >1 m/s for 6-meter walking distance, their physical capacity is considered as decreased; iii. assessment of skeletal muscle mass in the four extremities by Bioelectrical impedance analysis (BIA); if the Appendicular Skeletal Muscle Index of male patients is < 7.0 kg/m^2^ and that of female < 5.7 kg/m^2^, then the skeletal muscle mass of patients is deemed as reduced. Patients who conformed to i and iii or to ii and iii are confirmed with sarcopenia; those who met all three criteria above are considered as having severe sarcopenia.

Patients were ineligible for the study if they had infectious disease in the past 3 months, could not be able to follow treatment instructions due to cerebrovascular sequela or mental illness, had severe cardiopulmonary disease, liver disease or malignant tumor, or if they had received oral administration of corticosteroids or immunosuppressants in the last 6 months.

### Methods

The general and clinical data of patients including age, gender, hemodialysis duration, BIA-measured body mass and some lab indexes for the blood.

Specific interventions: for nutritional support, firstly a nutritional support team (NST), including one attending doctor or with higher titles, two specialty nurses in charge or in higher position, one pharmacist and one nutritionist, was organized. All team members ensured to be in possession of sufficient knowledge of their own specialty and also received additional NST training. During the treatment, the general and clinical data of patients were collected and assessed for their nutritional state, and factors might contribute to malnutrition were corrected. The doctors and pharmacist mainly accounted for assessing patients and making or adjusting treatment plans. The nutritionist guided each patient with their diet after their nutritional state evaluation. The two specialty nurses gave healthcare education to patients in accordance with their disease progression and physical condition, and supervised patients to record their daily food intake and stick to the nutritional plan made by the nutritionist. Patients were evaluated every 4 weeks for their nutritional state and to adjust their treatment plan accordingly, for a total of 12 weeks; for Baduanjin exercise, all enrolled patients were required to watch the video made by the State Sport General Administration of China, Baduanjin: A Health-care Qigong, twice to get familiar with the exercise. The video is about 20–30 min long. After that, patients were taught to do the exercise by a specialist to correct their moves for 2 weeks. Then they were instructed to do the exercise at their own home on non-hemodialysis days. The time and frequency of the exercise were designed according to previous reports (16), as 3 times a week, 2 h after each meal to do the exercise 2–3 times for 30–60min, for a total of 12 weeks. Patient 's vital signs were monitored closely during exercise. The exercise should be stopped immediately if any of the following circumstances, such as hypoglycemia, abnormal blood pressure, chest tightness, dizziness occurs. See Tables 1, 2 for specific exercise plans.

**Table 1:** Evaluation before exercise.

**Item**	**Contents**
Evaluation content and time	Detailed information collection: i. primary disease, vital signs and hemodialysis-related complications and the presence of other diseases etc.; ii. clinical indexes such as recent physical examination results and cardiopulmonary function assessment indexes; iii. hemodialysis details such as therapeutic plans, drug uses and vascular access and location etc. Basic exercise assessment: patient's regular exercise and physical capacity were evaluated once a week. Sarcopenia-related indexes assessment: the indexes including skeletal muscle mass and muscular strength were assessed once before and after Baduanjin intervention.
Assessment approaches	Medical evaluation results were obtained from analyzing patient's hemodialysis records and laboratory examination results. Basic exercise details were attained through questionnaires and communications with patients.
Contraindications	Exercise intervention was not quite fit for patients who had abnormal blood pressure (≥180/90mmHg or ≤ 90/60mmHg), severe cardiopulmonary diseases such as unstable angina, heart failure, valvular heart disease, aortic dissection, etc., poor pulmonary arterial hypertension control (>55mmHg on average), acute infectious diseases, who were not suitable for exercising due to osteoarticular diseases or open wounds in the body, had dry weight gain > 4kg because of hemodialysis and other complications that may be induced or aggravated by Baduanjin exercise.

**Table 2:** Baduanjin exercise plan.

Exercise plan	The whole process included warm-up, Baduanjin exercise and body relaxing. Warm up: 5 min for patients to stretch their body before the actual exercise; Baduanjin exercise: 30–60 min for patients to do the Baduanjin exercise. Body relaxing: patients were asked to massage and slightly pat their muscles to relieve their muscular tension.
Exercise time and frequency	The time and frequency of the exercise were set according to previous reports (16), as 3 times a week, 2 h after each meal to do the exercise 2–3 times for 30–60min, for a total of 12 weeks.
Exercise guidance and supervision	Patients were guided with correct Baduanjin exercise through professionals. Physical examination indexes of patients such as heart rate, respiratory frequency and blood pressure were monitored; Patients were observed for blood oozing in the vascular access site and swollen in the local skin during hemodialysis. Patients were observed for hypoglycemia-related symptoms such as perspiration, palpitation and hand tremors.
Exercise stopping signs	Patients should stop to take a rest or terminate Baduanjin exercise if any of the following circumstances occurs while exercising: i. presence of symptoms such as acute or difficult respiration and chest tightness; ii. presence of muscular spasm and joint pain; iii. abnormal blood pressure, heart rate and oxygen saturation of blood, which are manifested as blood pressure increasing over 180 mmHg, heart rate < 60 beats/min or oxygen saturation < 88%; iv. intolerant to over physical capacity consuming. If any of the circumstances occurs, the Baduanjin exercise should be stopped immediately for patients to take a rest or receive corresponding medications. If the discomfort continues after medication, patients should be sent to seek for professional medical treatment.

### Outcome measures

One-way analysis of variance and multivariate logistic regression were used to analyze the influencing factors of sarcopenia in patients undergoing MHD and the correlation of NLR with biochemical indicators (normal NLR is between 1 and 3, above 3 is deemed as increased). The value of NLR for diagnosing sarcopenia, and its correlation with patient's grip strength, gait speed and skeletal muscle mass indexes were explored, as well as influences of different interventions on the treatment of sarcopenia and on NLR.

### Statistical analysis

Data were analyzed using SPSS 22.0 software. Continuous variables were expressed as mean ± standard deviation (x ± sd). Data that did not conform to normal distribution were expressed as M (P25, P75), and those conforming to normal distribution and homogeneity of variance were analyzed by *t*-test and expressed as t. Data that did not conform to normal distribution and homogeneity of variance were analyzed with rank sum test and expressed as Z. Enumeration data were analyzed by Pearson chi-square test and expressed as chi-square. One-way analysis of variance was used for different variants, and binary logistic regression for detecting the risk factors of sarcopenia in MHD patients. ROC curve was used to analyze the value of NLR for diagnosing sarcopenia. Person test was used to analyze the correlation between two variables. *P* < 0.05 was considered statistically significant.

## Results

### Comparison among influencing factors for the occurrence of sarcopenia in MHD patients

Among the primarily enrolled 220 patients, 84 of them were diagnosed with sarcopenia (38.18%). The age, hemodialysis duration, the occurrence rate of coronary heart disease as well as the NLR of MHD patients with sarcopenia (sarcopenia group) were all higher than those without (non-sarcopenia group); and their serum albumin, hemoglobin, body mass index, grip strength, gait speed and skeletal muscle mass index were lower than those of MHD patients without sarcopenia (*P* < 0.05), as shown in Table 3.

**Table 3:** Comparison among influencing factors for the occurrence of sarcopenia in MHD patients.

**Item**	**Sarcopenia group (*n =* 84)**	**Non-sarcopenia group (*n =* 136)**	**χ^2^/*Z* value**	***P***
Male cases (%)	60 (71.43)	88 (64.71)	1.066	0.302
Age (year)	66.0 (58.0, 74.0)	59.0 (51.3, 68.0)	−3.783	< 0.001
Hemodialysis duration (month)	78.0 (68.0, 88.0)	64.0 (34, 5, 80.0)	−4.125	< 0.001
Education duration (year)	10.0 (5, 0, 11.0)	10.0 (7, 0, 11.0)	−1.457	0.145
Type 2 diabetes complication	32 (38.09)	46 (33.82)	0.414	0.520
Coronary heart disease complication	19 (22.62)	14 (10.29)	5.338	0.021
Hemodialysis thoroughness	1.67 (1, 43, 1.87)	1.61 (1, 44, 1.79)	−0.541	0.588
Serum albumin (g/L)	38.05 (34.43, 41.93)	41.50 (38.30, 42.50)	−3.501	< 0.001
Hemoglobin (g/L)	116.0 (89.0, 121.0)	118.0 (100.0, 122.0)	−1.972	0.044
Urea nitrogen before hemodialysis (mmol/L)	29.30 (19.80, 31.50)	29.55 (20.98, 31.50)	−0.423	0.672
Serum creatinine before hemodialysis (umol/L)	719.61 (373.31, 810.24)	728.62 (409.72, 810.24)	−0.443	0.665
Uric acid (umol/L)	478.50 (386.25, 497.00)	480.00 (392.50, 497.00)	−0.201	0.841
Triglycerides (mmol/L)	2.02 (1.59, 2.36)	2.19 (1.61, 2.36)	−0.588	0.556
Total cholesterol (mmol/L)	4.18 (3.23, 4.39)	4.22 (3.34, 4.39)	−0.418	0.676
High-density lipoprotein (mmol/L)	1.19 (0.75, 1.32)	1.22 (0.80, 1.32)	−0.513	0.608
Low-density lipoprotein (mmol/L)	1.67 (2.75, 3.88)	3.72 (2.87, 3.87)	−0.418	0.676
Serum potassium (mmol/L)	4.85 (3.89, 5.04)	4.88 (3.96, 5.05)	−0.128	0.898
Blood calcium (mmol/l)	1.85 (2.15, 2.22)	1.90 (2.16, 2.22)	−0.459	0.646
Serum phosphate (mmol/l)	2.09 (1.36, 2.23)	2.07 (1.46, 2.20)	−0.415	0.678
NLR	4.13 (2.57, 4.79)	2.57 (2.34, 3.30)	−4.849	< 0.001
PLR	116.87 (111.25, 136.21)	113.36 (110.54, 134.27)	−1.705	0.088
Body Mass Index (kg/m2)	23.38 (19.12, 24.56)	23.92 (21.50, 24.76)	−1.978	0.035
Grip strength (kg)	16.73 (12.84, 22.75)	31.64 (24.76, 33.58)	−9.914	< 0.001
Gait speed (m/s)	0.77 (0.43, 1.00)	0.94 (0.84, 1.02)	−3.731	< 0.001
Skeletal muscle mass index (kg/m^2^)	5.30 (5.10, 6.20)	7.25 (6.72, 7.40)	11.607	< 0.001

### Multivariate regression analysis of sarcopenia in MHD patients

Grip strength, gait speed and skeletal muscle mass index were excluded from the multivariate regression analysis because they were established diagnostic measurements for sarcopenia. However, by multivariate regression analysis, it was indicated that age, hemodialysis duration and NLR were independent factors contributing to the incidence of sarcopenia in MHD patients (see Table 4).

**Table 4:** Multivariate regression analysis of sarcopenia occurrence in MHD patients.

**Variables**	**β**	**SE**	**Wald value**	**OR value (95% CI)**	***P***
Constants	−0.482	0.139	12.056	–	0.001
Age (year)	0.042	0.015	7.381	1.043 (1.012–1.075)	0.007
Hemodialysis duration	0.022	0.006	11.833	1.022 (1.009–1.035)	0.001
NLR	0.441	0.142	9.621	1.555 (1.176–2.054)	0.002

### Correlation of NLR and albumin

**Figure 1:**
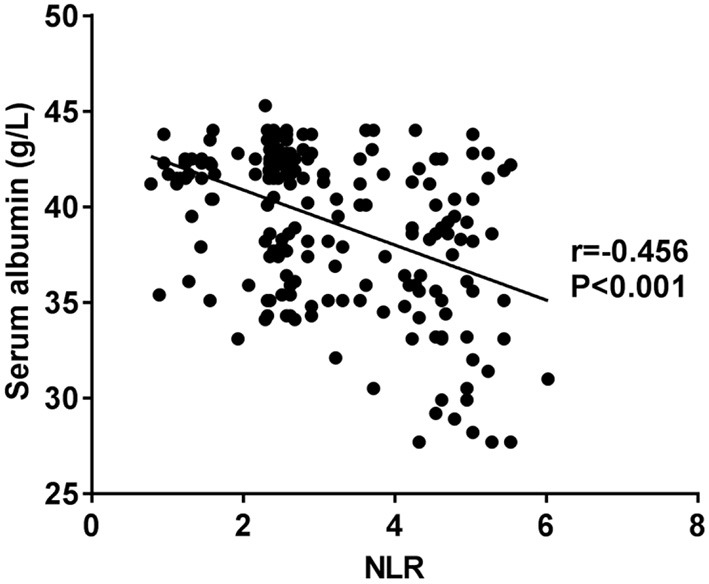
Correlation of NLR with albumin.

NLR is negatively correlated with serum albumin (see Figure 1).

### Diagnostic values of NLR for sarcopenia prediction in MHD patients

**Figure 2:**
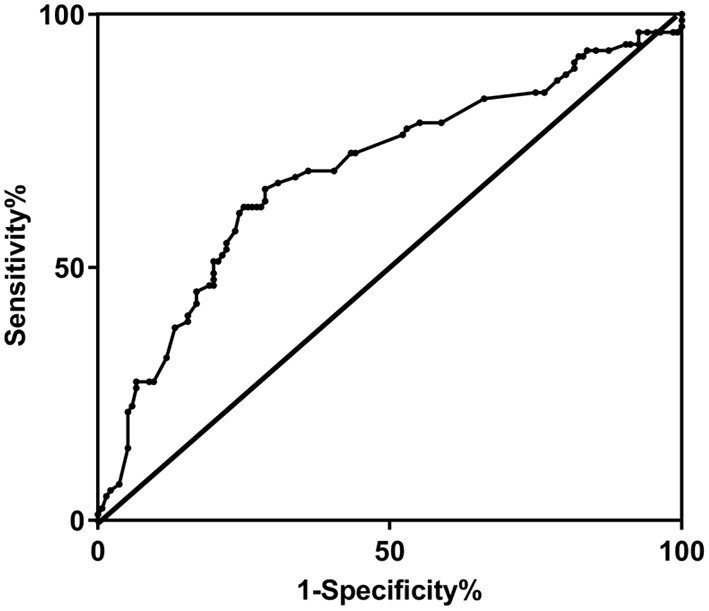
ROC curve of NLR for the diagnosis of sarcopenia in MHD patients.

The area under ROC curve of NLR for the diagnosis of sarcopenia in MHD patients was 0.695, and when NLR was at the 3.28 cut-off value, its Youden index was 0.369, specificity was 0.750 and sensitivity was 0.619, as shown in Figure 2.

### Correlation of NLR with grip strength, gait speed and skeletal muscle mass index

**Figure 3:**
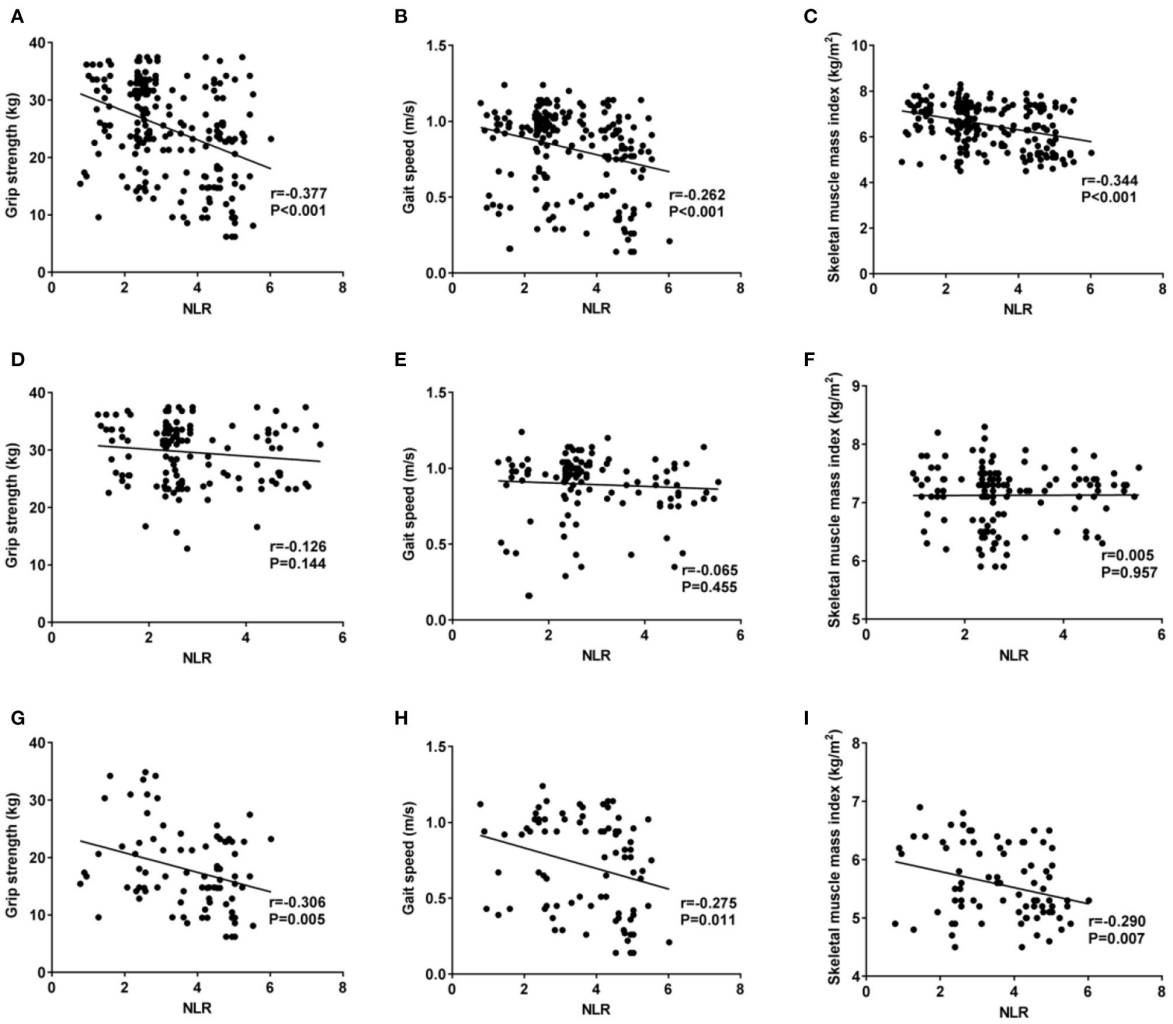
Correlations of NLR with grip strength, gait speed and skeletal muscle mass index, among which 3 **(A–C)** stand for correlations of NLR with grip strength, gait speed and skeletal muscle mass index of enrolled patients; **(D–F)** for correlations of NLR with grip strength, gait speed and skeletal muscle mass index of sarcopenia-free patients; and **(G–I)** for correlations of NLR with grip strength, gait speed and skeletal muscle mass index of sarcopenia patients. **(A, D, G)** show correlation of NLR with grip strength, **(B, E, H)** show that with gait speed, and **(C, F, I)** show that with skeletal muscle mass index.

NLR was negatively correlated with grip strength, gait speed and skeletal muscle mass index of enrolled patients (all *P* < 0.05), as shown in Figures 3A–C. No significant correlation was found between NLR and grip strength, gait speed and skeletal muscle mass index of patients without sarcopenia (all *P* > 0.05), as shown in Figures 3D–F. However, negative correlation was observed between NLR and grip strength, gait speed and skeletal muscle mass index of sarcopenia patients (all *P* < 0.05), as shown in Figures 3G–I.

### Comparison of baseline data between the two groups

No statistical differences were found in baseline data between the two groups (*P* > 0.05) (see Table 5).

**Table 5:** Comparison of baseline data between the two groups.

**Item**	**Observation group (*n =* 33)**	**Control group (*n =* 35)**	**χ^2^/*t*/*Z* value**	***P***
Male cases (%)	20 (60.61)	27 (77.14)	2.176	0.140
Age (year)	62.2 ± 10.0	62.1 ± 9.8	0.042	0.967
Hemodialysis duration (month)	78.0 (48.5, 99.2)	77.0 (70.0, 86.0)	−0.080	0.936
Education duration (year)	10.0 (5.0, 11.0)	9.0 (4.0, 11.0)	−0.285	0.775
Type 2 diabetes complication	13 (39.40)	14 (35.00)	0.003	0.959
Coronary heart disease complication	9 (27.27)	9 (25.71)	0.021	0.884
Hemodialysis adequacy	1.75 (1.35, 1.84)	1.71 (1.45, 1.83)	−0.443	0.658
Serum albumin (g/L)	40.4 (35.3, 42.5)	38.3 (34.8, 42.3)	−0.596	0.551
Hemoglobin (g/L)	118.0 (87.5, 121.0)	114 (86.0, 122.0)	−0.283	0.778
Urea nitrogen before hemodialysis (mmol/L)	27.3 (23.0, 31.2)	29.3 (19.8, 31.6)	−0.387	0.699
Serum creatinine before hemodialysis (umol/L)	637.5 (499.2, 785.5)	719.6 (361.5, 810.2)	−0.129	0.897
Uric acid (umol/L)	480.0 (419.5, 497.0)	486.0 (380.5, 505.0)	−0.129	0.897
Triglycerides (mmol/L)	2.01 (1.67, 2.28)	1.98 (1.57, 2.37)	−0.131	0.883
Total cholesterol (mmol/L)	4.01 (3.54, 4.37)	4.18 (3.23, 4.44)	−0.362	0.717
High-density lipoprotein (mmol/L)	1.09 (0.90, 1.29)	1.19 (0.75, 1.32)	−0.436	0.663
Low-density lipoprotein (mmol/L)	3.54 (3.06, 3.87)	3.67 (2.75, 3.92)	−0.424	0.672
Serum potassium (mmol/L)	4.88 (4.25, 5.04)	4.94 (3.82, 5.18)	−0.006	0.995
Blood calcium (mmol/l)	2.09 (1.95, 2.20)	2.15 (1.85, 2.24)	−0.393	0.694
Serum phosphate (mmol/l)	2.03 (1.45, 2.21)	2.09 (1.21, 2.23)	−0.227	0.820
NLR	3.48 ± 1.31	3.61 ± 1.21	0.427	0.670
PLR	116.8 (111.6, 136.8)	116.9 (111.2, 135.2)	−0.553	0.580
Body Mass Index (kg/m2)	23.92 (18.82, 24.56)	23.05 (18.43, 24.76)	−0.295	0.768
Grip strength (kg)	17.37 ± 7.60	19.29 ± 6.98	1.086	0.283
Gait speed (m/s)	0.75 ± 0.32	0.70 ± 0.30	0.685	0.497
Skeletal muscle mass index (kg/m^2^)	5.54 ± 0.62	5.53 ± 0.61	0.075	0.941

### Comparison of relevant indexes between the two groups before and after intervention

After interventions, grip strength and gait speed of patients in the observation group were higher than those in the control group, while the NLR was lower in the observation group than that in the control group (*P* < 0.05) (see Table 6).

**Table 6:** Comparison of relevant indexes between the two groups before and after intervention.

**Group**	**Cases**	**Grip strength (kg)**		**Gait speed (m/s)**		**Skeletal muscle mass index (kg/m** ^**2**^ **)**		**NLR**	
		**Before intervention**	**After intervention**	**Before intervention**	**After intervention**	**Before intervention**	**After intervention**	**Before intervention**	**After intervention**
Observation group	33	17.37 ± 7.60	21.38 ± 4.47	0.75 ± 0.32	0.91 ± 0.20	5.54 ± 0.62	5.62 ± 0.58	3.48 ± 1.31	2.96 ± 1.26
Control group	35	19.29 ± 6.98	18.37 ± 5.25	0.70 ± 0.30	0.71 ± 0.24	5.53 ± 0.61	5.58 ± 0.73	3.61 ± 1.21	3.59 ± 1.25
t value	-	1.086	2.539	0.685	3.654	0.075	0.312	0.427	2.093
P	-	0.283	0.013	0.497	0.001	0.941	0.756	0.670	0.040

## Discussion

As services and insurance policies in medical filed improve, an increasing number of patients have been able to receive MHD in China. However, in view of the specialty of MHD treatment and in consideration of the severeness of the disease requiring the treatment, patients have a high risk in getting sarcopenia, which in turn results in higher mortality and hospitalization rates, and increases burdens on hospitals as well as on patient's families (17). According to previous reports, the occurrence of sarcopenia in MHD patients reaches as high as 3.9–63.3% (18). In this study, 84 out of 220 (38.18%) patients were diagnosed with sarcopenia, conforming to the result mentioned above. Excluding the established diagnostic indexes for sarcopenia, it was found that age, hemodialysis duration and NLR were all independent risk factors contributing to sarcopenia in MHD patients. Why age is a contributing factor? Because the synthetic ability of testosterone in the body weakens with the increase of age, thus leading to decreased muscle mass because of reduced proteins in the muscles (19). One study suggested that patient's muscular strength and mass reduced year on year at a rate of 1–2% after the age of 50 years old. Also, the function of each organ declines as age increases, especially the function of stomach that hampers the absorption of nutrition, therefore increasing the risk of muscle loss (20). It has been shown that motor neuron loss is the main cause for sarcopenia. The motor neurons could be reduced with the growth of age, resulting in muscle fiber atrophy and degeneration, ultimately leading to muscle mass decline (21). A previous study suggested that hemodialysis duration was a major cause of sarcopenia (22). With the hemodialysis duration of patients getting longer, more patients tend to have nutrient loss, acid-base imbalance and hormone level changes, leading to an increase in the incidence of sarcopenia (4). Inflammation plays an important role in the development of sarcopenia as well. Several studies have shown that elevated levels of inflammatory factors could very likely contribute to decreased muscle mass and strength. Of these inflammatory factors, C-reactive protein (CPR), interleukin-6 (IL-6) and tumor necrosis factor-a (TNF-a) are critical for predicting the incidence of sarcopenia (23, 24). Micro-inflammation is a prevalent status in MHD patients due to the disease they have (25). NLR is an indicator reflecting systemic inflammatory state, which imbalances protein turnover and cell growth, thereby impairing skeletal muscle mass (13). Moreover, systemic inflammatory responses allow the body to produce more inflammatory factors, which could accelerate the breakdown of muscle proteins, leading to the occurrence of sarcopenia (26). Previous studies suggest that muscular strength in MHD patients could be decreased as contributions of many factors, among which malnutrition is critical one, leading to the onset of sarcopenia (27). Our one-way analysis of variance suggested that serum albumin was markedly decreased in MHD patients with sarcopenia, and multivariate regression analysis indicated that NLR was an independent risk factor for the occurrence of sarcopenia. And we further demonstrated that NLR was negatively correlated with albumin levels. Increased NLR might reduce albumin levels, resulting in sarcopenia caused by weakened skeletal muscles.

Our study showed that the area under ROC curve of NLR for predicting sarcopenia in MHD patients was 0.695. The specificity of NLR was 0.750 and the sensitivity was 0.619 at the cut-off value of 3.28. A study outside China that had included over 40,000 patients demonstrated that NLR was more advantageous and easier in assessing systemic inflammation in comparison with CRP (28). And a study in China suggested that high NLR might contribute to the occurrence of sarcopenia in middle age and elderly patients (29). Another cross-sectional study in Turkey showed that for every unit increase in NLR, elderly adults had a 1.31-fold increased risk of sarcopenia (30). Higher NLR was found to be associated with amyotrophy incidence in gastric cancer patients. The medium of NLR of gastric cancer patients diagnosed with amyotrophy was 3.15, with significantly lower 5-year survival rate of patients with high NLR and amyotrophy than those without (31). NLR is an abbreviation for neutrophil-to-lymphocyte ratio. It was reported that low lymphocyte level was related to malnutrition in elderly patients (32), and higher NLR is also related to malnutrition, leading to the occurrence of sarcopenia.

Currently, the occurrence of sarcopenia in MHD patients has gained attention in both China and other countries. However, the correlation of NLR with sarcopenia occurrence hasn't been studied, yet. In our study, it was concluded that for every unit increase in NLR, MHD patients had a 1.55-fold increased risk of sarcopenia. Grip strength, gait speed and skeletal muscle mass index are all clinical indicators for sarcopenia diagnosis, and also important measures reflecting muscle mass and strength. Our results showed that NLR was negatively correlated with grip strength, gait speed and skeletal muscle mass index, indicating NLR has certain value in predicting the incidence of sarcopenia.

Clinical interventions for MHD patients with sarcopenia have great significance for the prognosis and quality of life of patients, among which nutritional support and exercises are both important for the treatment of sarcopenia (33). At present, the nutritional support for MHD patients, i.e., oral administration of nutritional supplements, can effectively improve the synthetic and metabolic capacity of skeletal muscle proteins of patients (34). A study outside China showed that the nutritional support in addition to standard treatment for MHD patients could significantly improve their nutritional condition, and another study also suggested that the nutrition of patients who were inconvenient to have food regularly could be supplemented through additional nutritional support (35). Although nutritional support is effective in treating MHD patients with sarcopenia, such support should be customized for patients after thorough evaluation of their disease progression, body conditions and food-consuming habits. The nutritional support team is in charge of individualized supply of nutrition for patients who had malnutrition. It was reported that the team ought to make customized nutritional therapy for different patients according to their disease progression, diet habit and food intake amount, in order to enhance the compliance of patients (36). Exercises are also significant for MHD patients. The study of Tentori et al., which included 20,920 patients from 12 countries, observed the relation between physical exercise and the efficacy of hemodialysis, and found that doing physical exercise at least once a week could reduce the risk of death (37).

Baduanjin is an aerobic exercise with moderate intensity and easy operation, an exercise you can anytime in anywhere. A study suggested that doing Baduanjin exercise can improve the cardiopulmonary function in patients (38). It was reported that the physical conditions as well as quality of life of patients undergoing peritoneal dialysis had been improved after doing Baduanjin exercise for 12 weeks (39). It was also reported that for patients with chronic heart failure, their muscular strength in four extremities became prominently better after doing Baduanjin exercise for 24 weeks (40). As far as we know, no studies have yet reported the efficacy of Baduanjin exercise combined with nutritional support on sarcopenia treatment for MHD patients. And it is concluded in this study that the combined treatment can improve the muscular function of patients probably through alleviating the systemic inflammation in their bodies.

However, there are some limitations to this study. Firstly, the sample size is relatively small, a larger simple size should be designed for further exploration. Secondly, the intensity of Baduanjin can't be assessed effectively due to the lack of related tools. Thirdly, longer Baduanjin intervention period should be suggested to observe the prognosis of MHD patients with sarcopenia.

In summary, high NLR is a risk factor for the occurrence of sarcopenia, which has some certain value in predicting the incidence of the disease. However, sarcopenia in MHD patients can be treated through the employment of Baduanjin exercise in combination with nutritional support to enhance patient's muscular strength and reduce their systemic inflammation.

## Data availability statement

The original contributions presented in the study are included in the article/Supplementary material, further inquiries can be directed to the corresponding author.

## Ethics statement

The study was approved by the Ethics Committee of Nanjing Integrated Traditional Chinese and Western Medicine Hospital and Nanjing Hospital of Chinese Medicine (Ethics Approval Number 2021043). The patients/participants provided their written informed consent to participate in this study.

## Author contributions

JW, L-jH, and BL: played a critical role in conceptualizing this study, data analysis and interpretation, and review and revision of the manuscript. M-cX, LY, and XD: data collection. XD: draft manuscript. M-cX and LY: statistical analyses. All authors have read and agreed to the published version of the manuscript.
